# PASE: a novel method for functional prediction of amino acid substitutions based on physicochemical properties

**DOI:** 10.3389/fgene.2013.00021

**Published:** 2013-03-06

**Authors:** Xidan Li, Marcin Kierczak, Xia Shen, Muhammad Ahsan, Örjan Carlborg, Stefan Marklund

**Affiliations:** Division of Computational Genetics, Department of Clinical Sciences, Swedish University of Agricultural SciencesUppsala, Sweden

**Keywords:** PASE, amino acid substitution, physicochemical properties, functional prediction, mutation effect

## Abstract

**Background:** Non-synonymous single-nucleotide polymorphisms (nsSNPs) within the coding regions of genes causing amino acid substitutions (AASs) may have a large impact on protein function. The possibilities to identify nsSNPs across genomes have increased notably with the advent of next-generation sequencing technologies. Thus, there is a strong need for efficient bioinformatics tools to predict the functional effect of AASs. Such tools can be used to identify the most promising candidate mutations for further experimental validation.

**Results:** Here we present prediction of AAS effects (PASE), a novel method that predicts the effect of an AASs based on physicochemical property changes. Evaluation of PASE, using a few AASs of known phenotypic effects and 3338 human AASs, for which functional effects have previously been scored with the widely used SIFT and PolyPhen tools, show that PASE is a useful method for functional prediction of AASs. We also show that the predictions can be further improved by combining PASE with information about evolutionary conservation.

**Conclusion:** PASE is a novel algorithm for predicting functional effects of AASs, which can be used for pinpointing the most interesting candidate mutations. PASE predictions are based on changes in seven physicochemical properties and can improve predictions from many other available tools, which are based on evolutionary conservation. Using available experimental data and predictions from the already existing tools, we demonstrate that PASE is a useful method for predicting functional effects of AASs, even when a limited number of query sequence homologs/orthologs are available.

## INTRODUCTION

A non-synonymous single-nucleotide polymorphism (nsSNP) is a single-nucleotide change in a protein-coding region of a gene that causes an amino acid substitution (AAS) in the resulting protein. The importance of nsSNPs has been demonstrated in many studies (Risch and Merikangas, 1996; Collins et al., 1997; Chasman and Adams, 2001), and in databases such as the Online Mendelian Inheritance in Man (OMIM), AASs represent most of the genetic variants known to cause disease in human (Hamosh et al., 2005). An increasing use of next-generation sequencing (NGS) technologies to re-sequence genomes has resulted in an accelerated identification of nsSNPs.

As the functional effects of most AASs are experimentally unexplored, there is a need for development of tools to efficiently predict substitution effects on protein structure and function. Currently, several methods exist that are based on the degree of evolutionary conservation as an indicator of the functional effect of an AAS. Widely used software tools are, for example, SIFT (Ng and Henikoff, 2002, 2003) and PolyPhen (Stitziel et al., 2003, 2004). SIFT is based on sequence homology and position-specific scoring matrices with Dirichlet priors, whereas PolyPhen uses sequence conservation and ternary structure to model AAS sites combined with SWISS-PROT annotation (Ng and Henikoff, 2006). However, when the degree of sequence conservation in distant related species is difficult to assess due to a limited amount of orthologous sequences, alternative information such as physicochemical properties of amino acid (AA) may be applied for functional prediction of AAS. Here we present prediction of AAS effects (PASE), a novel method for efficient computational prediction and visualization of the effect of nsSNPs on the final protein. The prediction from PASE is based on the selected seven physicochemical properties (**Table 1**). The information obtained from PASE can also be combined with knowledge about sequence conservation to further improve functional predictions. Several examples are provided to illustrate the predictive ability of PASE both independently and combined with conservation information.

**Table 1:** Physicochemical properties of amino acids as described in the AAindex (Kawashima et al., 1999, 2008)

Descriptions	Terms from AAindex
Transfer of free energy from octanol to water	RADA880102
Normalized van der Waals volume	FAUJ880103
Isoelectric point	ZIMJ680104
Polarity	GRAR740102
Normalized frequency of turn	CRAJ730103
Free energy of solution in water	CHAM820102

## IMPLEMENTATION

**FIGURE 1:**
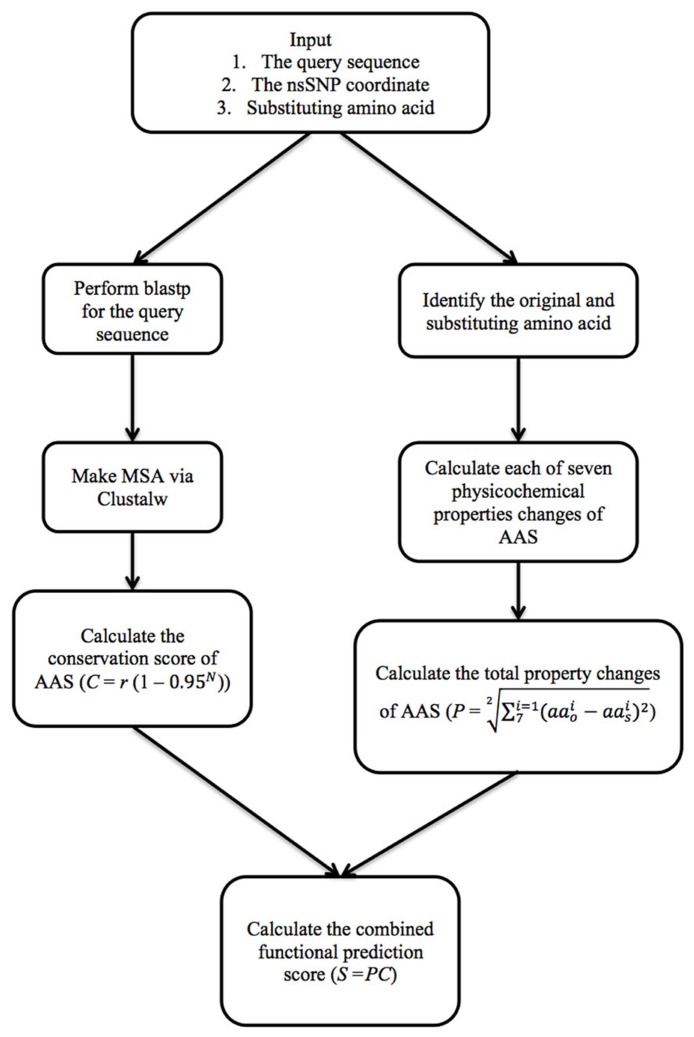
**The algorithm of PASE**.

The PASE algorithm uses physicochemical properties of AAs and sequence conservation to estimate the effect of each AAS on the protein (**Figure 1**). To calculate the AAS-induced change in the physicochemical properties, we imported seven physicochemical properties from the AAindex database previously selected by (Rudnicki and Komorowski (2004); **Table 1**), which gives every AA a unique profile. To illustrate how PASE can be combined with information about sequence conservation, we calculate a simple conservation score from a given protein sequence based on the alignment of homologous and orthologous sequences.

### PHYSICOCHEMICAL PROPERTY (PASE) SCORE

Each AA has a characteristic profile of physicochemical properties. Therefore, any AAS may influence the final protein structure by altering its physicochemical properties. PASE applies Euclidean distance formula to compute the physicochemical property changes of AAS between the original and the substituting AA:

(1) $$\begin{array}{c} P=\sqrt[{2}]{\Sigma_{7}^{i=1}\,{\left(a\,a_{o}^{i}-a\,a_{s}^{i}\right)}^{2},} \end{array}$$

where $a\,a_{o}^{i}$ and $a\,a_{s}^{i}$ indicate one of the seven physicochemical properties of original AA and substituted AA, respectively.

**FIGURE 2:**
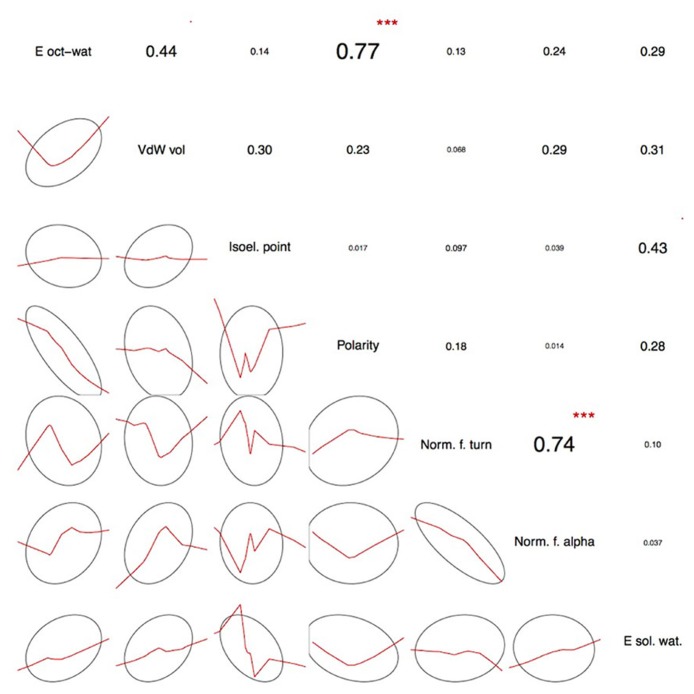
**Pairwise correlations between the selected physicochemical amino acid properties**. The actual correlation coefficients are presented in the upper triangle together with correlation significance symbols: ***p < 0.001, **p < 0.01, *p < 0.05, ’*p* < 0.1. Lower triangle shows smoothed trend lines together with confidence 1 SD ellipses. The vast majority of the selected properties show low pairwise correlation thus spanning a close-to-orthogonal coordinates frame in the physicochemical property space. As expected, there is a significant correlation between polarity-and transfer of free energy from octanol to water as the latter is to a large degree amino acid polarity-dependent. Similarly, normalized frequency of alpha-helix and normalized frequency of turn, the two secondary structure-related properties show significant pairwise correlation. This partial redundancy is the result of the physicochemical property selection procedure where the initial set of available AAindex descriptors has been narrowed down to only the easy-to-interpret properties. Thus, the selected seven properties are a reasonable trade-off between minimizing the number of dimensions necessary to preserve amino acid discernibility and the ease of interpretation. For a more detailed discussion, see Rudnicki and Komorowski (2004) and Kierczak et al. (2009).

**FIGURE 3:**
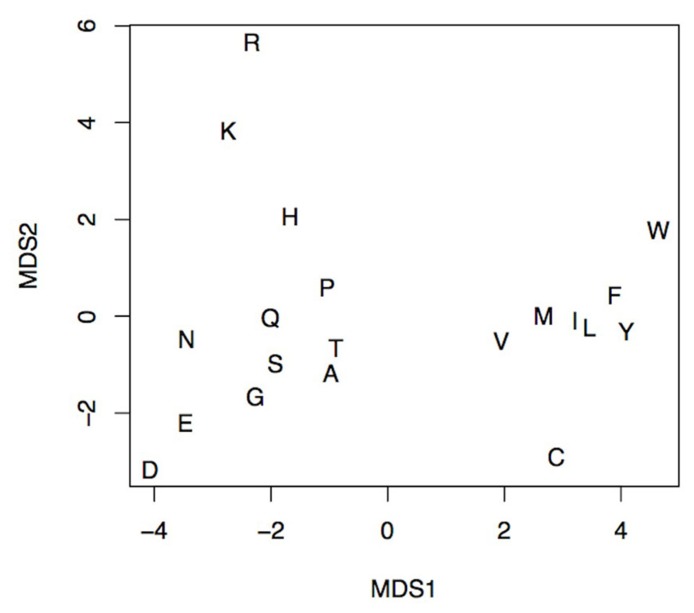
**An MDS projection of the AA’s property space spanned by the seven selected physicochemical properties**. The distance between any pair of letters corresponds to the physicochemical similarity between respective amino acids. The figure shows the ability to discern any pair of amino acids using selected physicochemical properties. The more similar amino acids, the smaller the distance between them, e.g., Ile (I) and Leu (L). Likewise, amino acids with similar physicochemical properties like basic Lys (K), Arg (R), and His (H) form distinct groups.

The seven physicochemical properties: (1) transfer of free energy from octanol to water, (2) normalized van der Waals volume, (3) isoelectric point, (4) polarity, (5) normalized frequency of turn, (6) normalized frequency of alpha-helix, and (7) free energy of solution in water used by PASE, have been selected from the AAindex database of protein indices (Kawashima et al., 1999, 2008) using an algorithm described by Rudnicki and Komorowski (2004). Briefly, the properties were selected to reflect the major biologically meaningful features of protein sequences: (1) hydrophobicity, (2) polarity, (3) size, (4) tendency to form particular secondary structure, and (5) electrostatic properties of the AA. At least one property has been selected from each of these broad groups and resulted in a seven tuple of properties. Due to the low pairwise correlation within the selected properties, the ability to uniquely identify each AA is preserved (Kierczak et al., 2009; **Figure 2**). In other words, the selected properties span a nearly orthogonal 7D coordinates space in which every AA is a unique point (**Figure 3**).

### MSA CONSERVATION SCORE

Highly conserved sequences often indicate a functional importance of the AA and substitutions tend to be deleterious, whereas those within areas of low conservation are often tolerated. We calculate a simple sequence conservation score (multiple sequence alignment conservation, MSAC), by searching for functionally related protein sequences by using NCBI-blastp (Thompson et al., 1994) and generating an alignment with multiple homologous sequences with an arbitrary threshold of 0.01, where the redundant homologous sequences are filtered out in order to retrieve only one hit per species. ClustalW (Larkin et al., 2007) is used to make a multiple sequence alignment (MSA) to score conservation at each site. To accomplish it, we use the following formula:

(2) $$\begin{array}{c} C=r\,\left(1-0.95^{N}\right), \end{array}$$

where *N* is the number of assessed sequences of MSA, and *r* is the proportion of the AAs of interest in MSA. The formula (1 - 0.95^*N*^; Pei and Grishin, 2001) indicates the probability of 20 different AAs in a position for *N* random equal frequent AA sequences. For example, when *N* = 1, the probability of each AA in a position is 0.05, which is consistent with 20 AAs with frequency 1/20 each.

### COMBINED PASEC SCORE

The PASE score can be combined with information about sequence conservation by creating a combined score – PASEC. PASEC is computed as *S* = *PC*, where the score of physicochemical properties changes is multiplied by the conservation score. The PASEC score ranges from 0 to 1, where 0 is neutral, and higher ratio indicate stronger predicted effects on the protein. We have here for illustration combined PASE with a simple MSAC score, but other and more advanced measures of conservation can be used in the same manner.

## RESULTS AND DISCUSSION

To explore the predictive ability of the new method, we have studied two protein sequences, with known AASs that have well-characterized phenotypic effects.

### nsSNPs IN THE HUMAN *Cx50* GENE

Gap junction proteins, also called connexins, belong to a family of channel-forming structure proteins in contacting plasma membranes (Dbouk et al., 2009). Recently, two consecutive AASs (W45S and G46V) in the human connexin 50 (*Cx50*) gene have been associated with cataracts. It has been shown that these two mutations cause the disease through different mechanism (Tong et al., 2011).

As shown in **Table 2**, both W45S and G46V show large properties changes (PASE scores 0.80 and 0.56) as well as high conservation scores (MSAC scores for both of 0.93). The combined PASEC scores are 0.74 and 0.52 for W45S and G46V, respectively, indicating considerable expected effects on protein function. This is consistent with the disease association of both these AASs (Tong et al., 2011) as well as with predictions made using the SIFT and PolyPhen tools, although the PolyPhen prediction were less decisive regarding the effect of the mutations (**Table 2**).

**Table 2:** Functional prediction of AASs in Cx50 and PRKAG3.

Genes	AAS	MSAC score	PASE physicochemical score	PASEC score	SIFT	PolyPhen
Cx50	W45S	0.93	0.8	0.74	Deleterious	Possibly damaging
	G46V	0.93	0.56	0.52	Deleterious	Possibly damaging
PRKAG3	I199V	0.93	0.14	0.12	Tolerated	Benign
	R200Q	0.85	0.54	0.5	Deleterious	Probably damaging

### nsSNPs IN THE PORCINE *PRKAG3* GENE

The protein kinase AMP-activated gamma 3 (*PRKAG3*) gene encodes the regulatory γ subunit of adenosine monophosphate-activated protein kinase (AMPK), which is prevalently expressed in white skeletal muscle fibers (Mahlapuu et al., 2004). The dominant *RN* mutation that causes excess glycogen content in pig skeletal muscle is caused by the R200Q AAS in AMPKγ3 of purebred Hampshire pigs (Enfält et al., 1997; Milan et al., 2000). A substitution of the adjacent AA, I199V accounts for smaller increase in the muscle glycogen content than the R200Q substitution, but also co-participates with R200Q in the process (Ciobanu et al., 2001).

Exploration of the conservation and changes in physicochemical property of the AASs in the porcine PRKAG3 revealed large physicochemical property changes for R200Q (PASE score 0.54) and smaller for I119V (PASE score 0.14) as well as high degrees of conservation at both 199th (MSAC score 0.85) and 200th (MSAC score 0.93) sites (see **Table 2**). The combined PASEC functional prediction scores are 0.50 and 0.12, respectively, thus consistent with the observed phenotypic effects and with the SIFT and PolyPhen predictions for these mutations (see **Table 2**).

### COMPARISON OF FUNCTIONAL PREDICTION SCORES FOR PASE, PASEC, SIFT, AND PolyPhen

**FIGURE 4:**
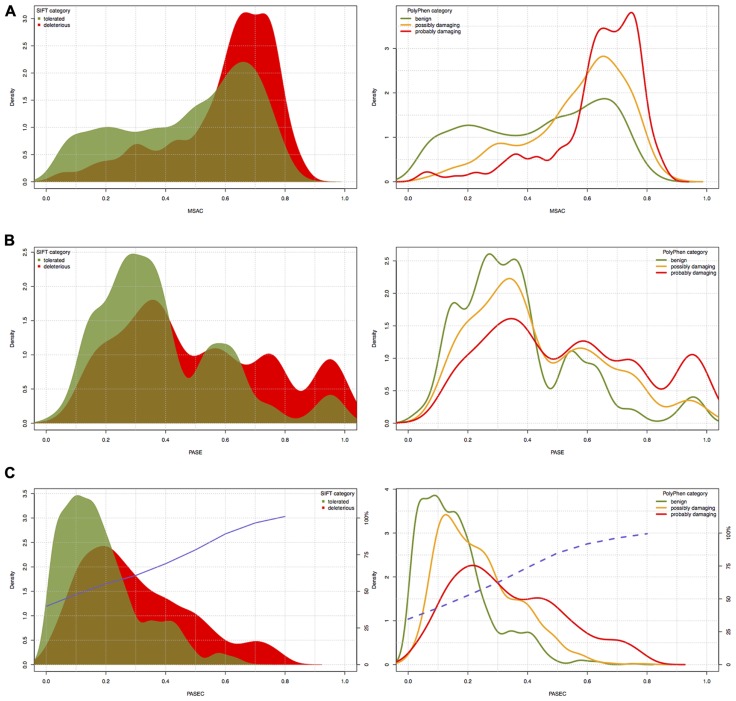
**The distribution of (A) PASE, (B) MSAC, and (C) PASEC scores within different SIFT and PolyPhen prediction classes**. Blue solid and dashed lines in panel **(C)** correspond to the probability of deleterious/damaging prediction from AAS’s PASEC scores.

To further evaluate the PASE and PASEC predictions, we used 3338 AASs in the Ensembl database representing nsSNPs on human chromosome 22 with functional effects previously predicted with the widely used SIFT and PolyPhen tools. For AASs predicted with SIFT as “tolerated” (1987 AASs) or with PolyPhen as “benign” (1637 AASs), PASE/PASEC showed average scores of X/0.18 and Y/0.16, respectively, whereas AASs predicted with SIFT as “deleterious” (1351 AASs) or with PolyPhen as “probably damaging” (1162 AASs) showed average scores of Z/0.3 and A/0.33 (**Table 3**). The distributions of PASE, MSAC, and PASEC scores are shown in **Figures 4A–C**, respectively.

**Table 3:** Functional predictions of AASs in Human Chromosome 22.

Name of tools	Classifications	Number of AAS	MSAC score	PASE physicochemical score	PASEC scores
SIFT	Tolerated	1987	0.47	0.39	0.18
	Deleterious	1351	0.6	0.51	0.3
PolyPhen	Benign	1637	0.44	0.37	0.16
	Possibly damaging	539	0.56	0.43	0.24
	Probably damaging	1162	0.63	0.53	0.33

The PASE is novel in its approach to utilize seven physicochemical properties of AA’s to predict the effect of an AAS. In **Figure 4**, we illustrate the overlap between the scores obtained with PASE, MSAC, and PASEC and those obtained using the conservation-based scores from SIFT and PolyPhen. **Figure 4A** shows that the PASE score provides novel predictions regarding which AAS are likely to have a functional effect in that both AAS that have high and low SIFT and PolyPhen scores have high PASE scores. Interestingly, however, there is an overrepresentation of AASs predicted to be deleterious/damaging among those with high PASE scores, which indicates that combining information on conservation and physicochemical properties might help to further refine the predictions of function of AAS. **Figure 4B** shows the overlap between the simple conservation score MSAC and SIFT/PolyPhen. The MSAC score overlaps to a large extent with the predictions of deleterious/damaging AAS from SIFT/PolyPhen, in that such AAS have higher MSAC scores, but shows little overlap for the tolerated/benign AAS. **Figure 4C** shows that the combined PASEC score, combines the information about the physiochemical properties in the PASE score and the conservation information in the MSAC score to further refine the predictions. As shown in **Table 4**, at PASEC scores above 0.8, all of these are predicted by SIFT/PolyPhen to be damaging/deleterious and at a score of 0.5 this has decreased to 81/87%. Most of the AASs with high PASEC scores are thus defined as deleterious/damaging in SIFT/PolyPhen. There are, however, also a few AAS defined as tolerated/benign with high PASEC scores and a large number of AAS defined as deleterious/damaging with low PASEC scores. This probably reflects that at poorly conserved locations AASs often do not affect the protein function significantly, and even large physicochemical property changes are tolerated and more easily become abundant. Conversely, at highly conserved locations the protein function is very sensitive even for AASs with small physicochemical property changes. Combining conservation and physicochemical property scores thus identifies a smaller number of functional candidates that simultaneously fulfills both criteria and thus are the most interesting candidates for further characterization. We have here used MSAC as a simple score for measuring sequence conservation and compared the overlap to results from other methods based on sequence conservation. It should be noted that MSAC only serves an illustrative purpose in our description of PASEC and could also be exchanged by sequence conservation scores from SIFT or PolyPhen when applied to real data.

**Table 4:** Proportion of AAS’s with deleterious/damaging predictions from the SIFT/PolyPhen algorithm at different PASEC scores.

PASEC score range	SIFT (%)	PolyPhen (%)
0.8	100	100
0.7	100	95
0.6	86.50	91
0.5	80.70	86.60
0.4	67.10	71.60
0.3	61.40	62.70
0.2	55.70	53.30
0.1	47	42.10
0	40	34.80

Our future research will focus on tailoring PASEC to specific protein classes (e.g., *trans*-membrane proteins) and protein families. We are planning to use statistical learning methods to take into account the specific microenvironment of the substituted AA and to infer more general, interpretable rules describing the degree of AAS impact on protein function. To this end we need to collect larger datasets of carefully curated examples of different classes of AAS and to integrate information available from several sources, like databases, structure prediction servers, etc.

## CONCLUSION

Functional prediction of AASs is highly important in genetic studies. Functional importance of an AA is often reflected by a high degree of evolutionary conservation. This has earlier been used for functional prediction of AAS in, e.g., the SIFT and PolyPhen algorithm predictions. Here, we propose a new method, PASE, which predicts the functional effect of an AAS based on the physicochemical properties of the alternative AAs. This allows prediction of the potential effect of an AAS in the protein structure and its interaction with other residues. PASE complements already available tools by allowing such functional prediction of an AAS’s effect in situations where information on evolutionary conservation is not available or applicable. As the physiochemical property of an AA and its degree of evolutionary conservation are independent, these two sources of information should if combined be able to better predict the potential functional importance of an AAS. Here, we also show the usefulness of this combined approach and its potential to refine predictions made by the approaches independently.

## AVAILABILITY AND REQUIREMENTS

**Project homepage:** http://www.computationalgenetics.se/wp-content/uploads/software/PASE/pase.tar

**Project name:** PASE

**Operating system(s):** Linux and Unix

**Programing language:** Python and Biopython

**Other requirements:** Python 2.6 or higher, Biopython 1.58 or higher and ClustalW (1.82) or higher

**License:** GNU

**Any restrictions to use by non-academics:** none

## AUTHOR CONTRIBUTIONS

Marcin Kierczak initiated the study. Marcin Kierczak, Xidan Li, Stefan Marklund, and Örjan Carlborg planned the study. Xidan Li, Marcin Kierczak, Xia Shen, Stefan Marklund, and Örjan Carlborg developed the algorithm that was implemented by Xidan Li and Marcin Kierczak. Xidan Li and Muhammad Ahsan evaluated the algorithm. Xidan Li and Marcin Kierczak drafted the manuscript and all authors contributed to the final version of the manuscript.

## Conflict of Interest Statement

The authors declare that the research was conducted in the absence of any commercial or financial relationships that could be construed as a potential conflict of interest.

## Abbreviations

AA: amino acid
AAS: amino acid substitution
MDS: multiple dimensional scaling
MSA: multiple sequence alignment
MSAC: MSA conservation
nsSNP: non-synonymous single-nucleotide polymorphism
PASE: prediction of amino acid substitution effects
PRKAG3: protein kinase AMP-activated gamma 3
SNP: single-nucleotide polymorphism.
